# Research progress on sirtuin family genes in colorectal cancer

**DOI:** 10.3389/fonc.2025.1570030

**Published:** 2025-09-23

**Authors:** Tong Liu, Tianhua Wu, Lijun Luo, Wei Li, Minglin Zhang

**Affiliations:** ^1^ Department of General Surgery, Zhongshan Hospital of Traditional Chinese Medicine Affiliated to Guangzhou University of Traditional Chinese Medicine, Zhongshan, Guangdong, China; ^2^ School of Medical Laboratory Science, Hebei North University, Zhangjiakou, Hebei, China; ^3^ Department of Gastroenterology, The Third Xiangya Hospital, Central South University, Changsha, Hunan, China

**Keywords:** sirtuin family, colorectal cancer, metabolic reprogramming, epigenetic regulation, targeted therapy

## Abstract

Sirtuins are a highly conserved family of NAD^+^-dependent deacetylases involved in regulating critical biological processes such as cell survival, antioxidation, gene transcription, proliferation, differentiation, DNA repair, and mitochondrial function. Recent studies have revealed that altered expression of sirtuin family genes in mammals is closely linked to the development of various diseases, including metabolic disorders, ageing, and cancer. In colorectal cancer (CRC), sirtuins play dual regulatory roles, modulating tumour cell proliferation, migration, invasion, and apoptosis while also activating pro-oncogenic signalling pathways or suppressing tumour progression, depending on context. This review systematically summarizes the research progress on sirtuin family genes in CRC, highlighting their dual roles (pro-tumorigenic and tumour-suppressive) and molecular mechanisms. These findings underscore the potential of sirtuins as therapeutic targets in CRC. The development of selective activators or inhibitors, combined with metabolic interventions or immunotherapy, may provide novel strategies for precision CRC treatment.

## 1 Introduction

Colorectal cancer (CRC) is the third most common malignant tumour worldwide and a major global health threat, accounting for 10% of cancer-related deaths in high-income countries. Survival rates range from 50–93%, depending on the tumour stage (1). Patients with early-stage CRC often lack obvious symptoms such as bleeding, abdominal pain, or weight loss, leading to a diagnostic delay of approximately 7–10 years or even decades. The 5-year survival rate is significantly lower in patients with advanced CRC than in those diagnosed at an early stage (1, 2). Most patients with CRC are diagnosed at advanced stages when metastasis has already occurred (3). Therefore, early colonoscopy screening is critical. Globally, epidemiological factors such as age, obesity, smoking, and alcohol consumption increase the risk of CRC development (4). The development of CRC involves complex genetic mutations and dysregulation of pathways. During the progression from normal mucosa to adenoma and then to adenocarcinoma, mutations in core driver genes and dysregulation of pathways accumulate progressively. Among these, alterations in the APC/β-catenin pathway represent the earliest critical event initiating adenoma formation (5). Inactivation of the APC gene impairs the degradation of β-catenin, leading to its nuclear translocation and persistent activation of the Wnt pathway, thereby promoting tumorigenesis (5). Familial adenomatous polyposis (FAP) exemplifies this mechanism and is primarily caused by germline mutations in the APC gene (6). Studies in Chinese populations indicate that approximately 60% of FAP patients carry APC missense mutations, without intervention, their risk of cancer transformation by age 40 is extremely high (7).

Beyond APC mutations, CRC progression involves mutations in other key genes such as KRAS, BRAF, TP53 and PIK3CA (8, 9). Microsatellite instability (MSI) is also a significant molecular feature. MSI arises from deficient DNA mismatch repair (MMR) function, leading to the accumulation of uncorrected errors in microsatellite sequences during DNA replication. MMR deficiency is typically caused by pathogenic mutations in MLH1, MSH2, MSH6, PMS2, or EPCAM genes (10–12). Based on the degree of instability, MSI is classified into three categories: high instability (MSI-H), low instability (MSI-L), and stable (MSS) (11). A large UK cohort study further indicated that MMR gene mutations contribute to up to 10.9% of familial CRC cases. Additionally, mutations in genes such as POLE/POLD1, STK11, and MUTYH have also been confirmed to increase CRC risk (13). Beyond these classic pathways and gene mutations, gene-gene interactions have also been found to contribute to CRC risk. For example, genome-wide association studies revealed a significant interaction between the missense variant rs138649767 in TCF7L2 and the regulatory variant rs6983267 in the MYC enhancer region (14). Furthermore, abnormal epigenetic regulation plays a crucial role in CRC. Mutations in ARID1A, an important epigenetic regulator, have been found to be associated with acquired resistance to cetuximab treatment (15). Tumour metabolic reprogramming and epigenetic modifications, through the coordinated action of key genes and epigenetic regulators, modulate signalling pathways and gene expression to collectively drive the malignant progression of CRC (16). The development of CRC is a multifactorial, multi-step complex process driven by the coordinated action of multi-layered molecular events.

Sirtuins are NAD^+^-dependent enzymes with multifaceted catalytic capabilities, encompassing desuccinylation, demalonylation, deglutarylation, deformylation, and depalmitoylation activities (17, 18), the histones and non to acetylation of histone lysine residues and NAD^+^ hydrolysis combined with, and NAD^+^-dependent deacetylases that regulate histone proteins at specific lysine residues, promoting posttranslational modifications that result in chromatin silencing and transcriptional repression (19–21). Sirtuin family genes relate sirtuin activity to energy metabolism by relying on the cell-level coenzyme NAD^+^, which is produced through two different biological pathways. The hydride receptor NAD^+^ is produced from scratch by the ingestion of essential amino acids in the diet (22). NAD^+^ is a precursor of NADP^+^/NADPH, which is necessary for cell biosynthesis pathways to protect cells from reactive oxygen species (ROS) (22). Therefore, NAD^+^ plays key roles in the regulation of redox status and energy metabolism, and sirtuin family genes regulate energy metabolism by binding NAD^+^ to deacetylate key lysine residues of metabolic proteins.

In mammals, seven sirtuin family genes (SIRT1–7) are expressed and constitute an evolutionarily conserved family of enzymes involved in different but related physiological processes (20). In brief, SIRT1–7 target different acetylated protein substrates and are located in different subcellular compartments. SIRT1 is located mainly in the nucleus, but has also been detected in the cytoplasm (23). SIRT2 was originally described as a cytosolic protein; however, recent data show that SIRT2 is also expressed in the nucleus, where it functions to modulate cell cycle control (24). SIRT3, SIRT4 and SIRT5 are localized in mitochondria and are involved in ATP production, metabolism, apoptosis and the regulation of cell signalling pathways (25, 26). SIRT6 and SIRT7 are present in the nucleus, where they function to deacetylate histones, thereby influencing gene expression epigenetically (27).

**Figure 1:**
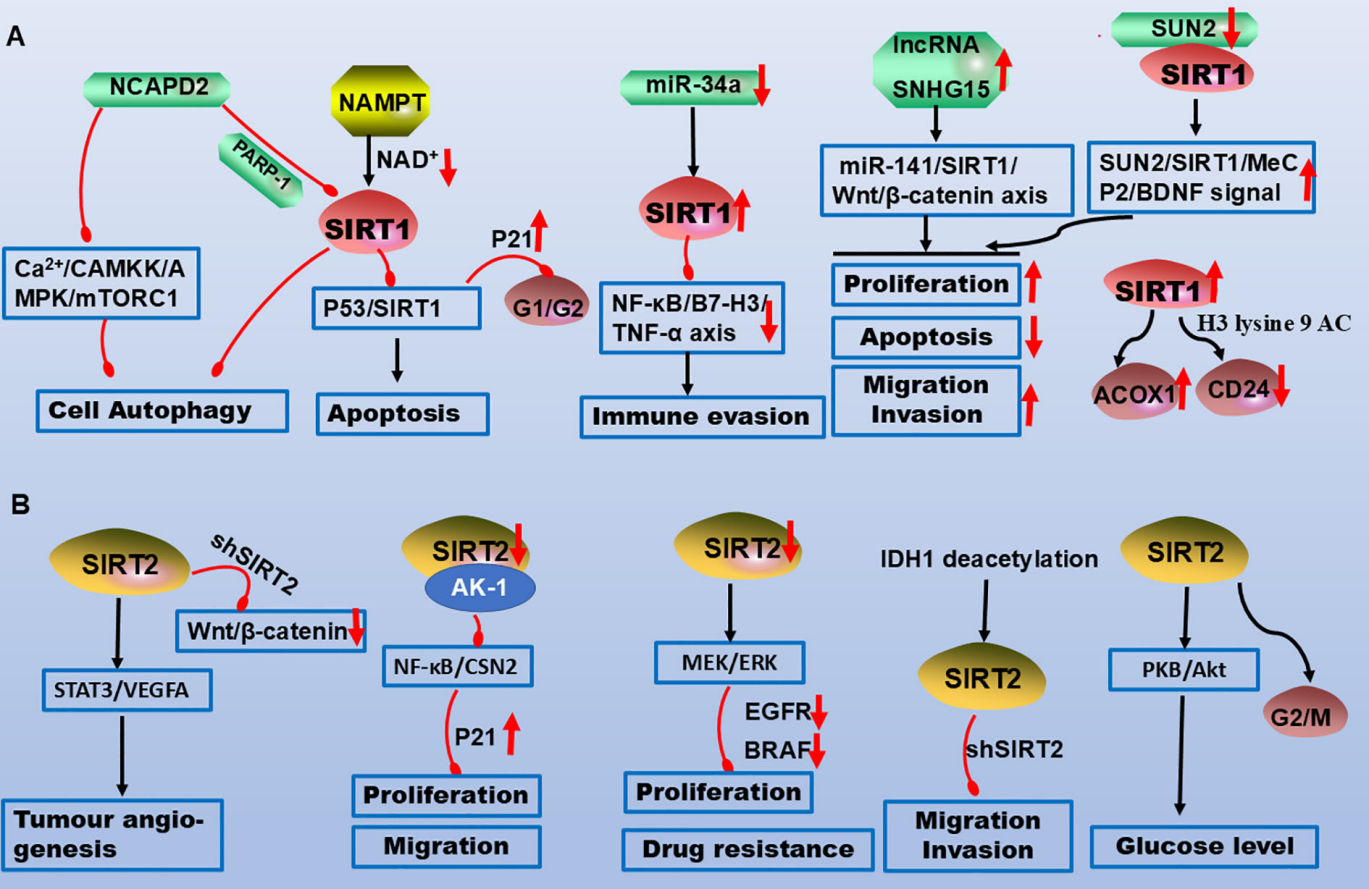
Molecular mechanisms of SIRT1 and SIRT2 in CRC pathogenesis. **(A)** NCAPD2 inhibits SIRT1, promoting apoptosis and cell cycle arrest. Downregulation of miR-34a upregulates SIRT1, which suppresses the NF-κB/B7-H3/TNF-α axis to facilitate immune evasion. The lncRNAs SNHG15 and SUN2 interact with SIRT1 to promote CRC proliferation, migration, and invasion while inhibiting apoptosis. **(B)** SIRT2 promotes angiogenesis and glucose metabolism via STAT3 and AKT signalling. Downregulation of SIRT2 inhibits the NF-κB and MEK/ERK pathways, suppressing proliferation, migration, and chemoresistance. Legend: Red upwards arrows: Target upregulation or activation; Red downwards arrows: Target downregulation or inhibition; Black arrows: Promotion or activation; Red blunt ends: Inhibition.

**Figure 2:**
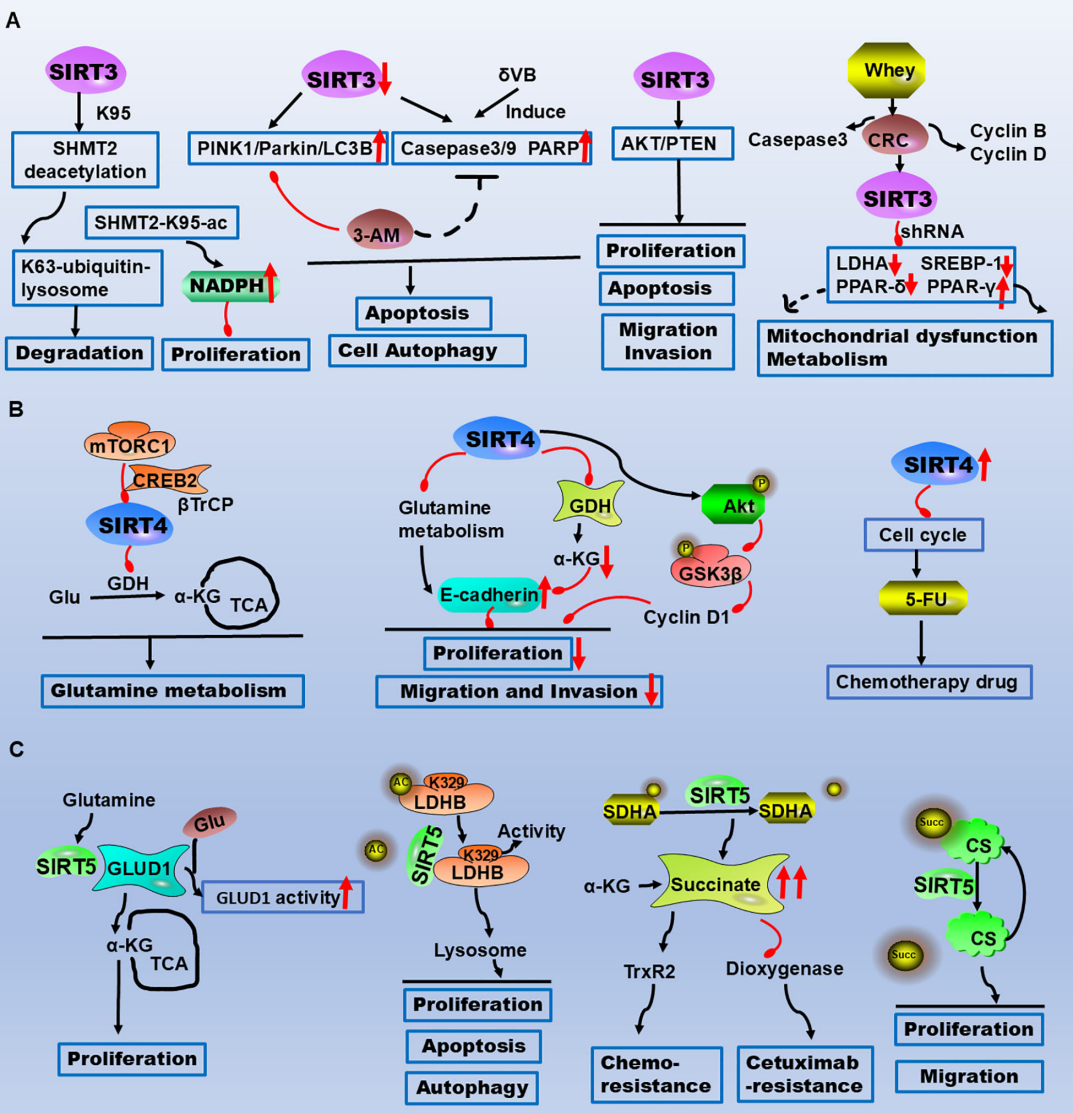
The critical roles of SIRT3/4/5 in CRC metabolism, apoptosis, and therapeutic resistance highlight their potential as therapeutic targets. **(A)** SIRT3 reduces CRC cell proliferation and tumour growth by deacetylating SHMT2 at the K95 site. δVB downregulates SIRT3, induces mitophagy, and triggers apoptosis via the PINK1/Parkin/LC3B axis. SIRT3 regulates metabolic reprogramming via PPAR-α, PPAR-γ, and PPAR-δ. **(B)** mTORC1 regulates glutamine metabolism by inhibiting SIRT4 to control GDH activity. SIRT4-dependent glutaminase suppresses proliferation, migration, and invasion through the AKT/GSK3β/CyclinD1 pathway. SIRT4 inhibition increases the sensitivity of CRC cells to the chemotherapeutic drug 5-FU by arresting the cell cycle. **(C)** SIRT5 directly interacts with GLUD1, leading to its deglutarylation and activation, thereby promoting proliferation. SIRT5 induces LDHB deacetylation at the K329 site, increasing LDHB enzyme activity to regulate autophagy, apoptosis, and proliferation in CRC cells. SIRT5 demalonylates and inactivates SDHA, causing the accumulation of the oncometabolite succinate. Succinate binds to and activates the reactive oxygen species (ROS)-scavenging enzyme TrxR2, conferring resistance to chemotherapy. Legend: Red upwards arrows: Upregulation or activation of the target. Red downwards arrows: Downregulation or inhibition of the target. Black arrows: Promotion or activation. Red blunt ends: Inhibition.

**Figure 3:**
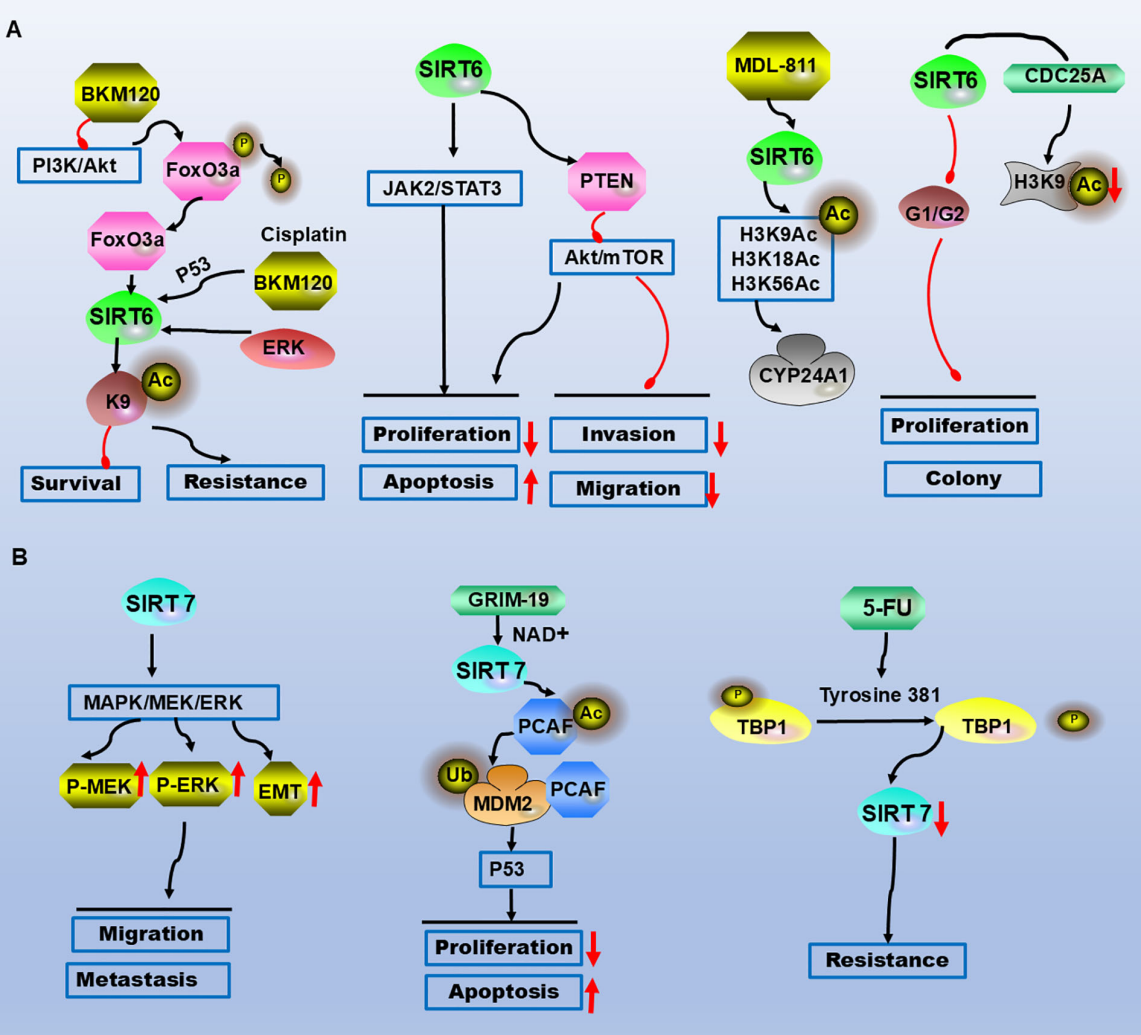
SIRT6 and SIRT7 are involved in the molecular mechanisms of CRC occurrence and development. **(A)** AKT is inhibited, leading to FoxO3a dephosphorylation and its binding to the SIRT6 promoter, contributing to drug resistance and survival. SIRT6 suppresses proliferation, migration, and invasion via the JAK2/STAT3 and AKT/mTOR pathways while promoting apoptosis. The selective SIRT6 activator MDL-811 activates SIRT6-mediated deacetylation of histone H3 (H3K9Ac, H3K18Ac, H3K56Ac) *in vitro*, targeting the downstream gene CYP24A1. SIRT6 induces G0/G1 phase arrest and binds to the CDC25A promoter, reducing histone H3 lysine 9 acetylation to inhibit proliferation and clonogenicity. **(B)** SIRT7 promotes cell migration and metastasis through the MEK/ERK/MAPK signalling pathway and EMT. GRIM-19 inhibits CRC cell proliferation and induces apoptosis in a p53-dependent manner via the SIRT7/PCAF/MDM2 axis. Upon 5-FU treatment, TBP1 targets SIRT7 through a ubiquitin-independent and proteasome-dependent pathway, leading to chemoresistance. Legend: Red upwards arrows: Upregulation or activation of the target. Red downwards arrows: Downregulation or inhibition of the target. Black arrows: Promotion or activation. Red blunt ends: Inhibition.

Sirtuin family genes also regulate the lifespans of yeast, nematodes, fruit flies and mice (28, 29). Changes in sirtuin expression are associated with a variety of diseases and have become relevant factors for metabolic regulation, including metabolism, diabetes, cardiovascular disease, cancer and ageing (30). In recent years, several studies have shown that seven sirtuin family genes are involved in CRC development. In this review, we focus on the biological functions of various sirtuin family genes and their roles in CRC pathophysiology (**Table 1**, **Figures 1**–**3**).

**Table 1:** Basic characteristics of the sirtuin family genes and its role and molecular mechanism in CRC.

Sirtuins	Localization	Chromosomal /aa	Enzymatic activity	Role and molecular mechanism in CRC	Reference
SIRT1	Nucleus, cytoplasm	747 aa	Deacetylation	Regulates proliferation, invasion and metastasis of CRC.	(39, 44)
				Activates the role of SIRT1- miR-1185-1-CD24 axis in CRC.	(45)
				Through NF-κB, P53 and Wnt/β-catenin signaling induce CRC.	(46, 53)
SIRT2	Nucleus, cytoplasm	Chromosome 19, containing 18 exons	Deacetylation, Demyristoylase	SIRT2 regulates blood glucose levels through PKB/AKT signaling.	(66)
				SIRT2 is involved in proliferation, migration and invasion.	(69)
				Through Wnt/β-catenin and RAS/RAF/MEK/ERK signaling induce CRC.	(74)
SIRT3	Mitochondria	257 aa	Deacetylation	Regulates proliferation, invasion, metastasis and apoptosis of CRC.	(83)
				SIRT3 via AKT/PTEN, PINK1/Parkin/LC3B axis, PPAR-α, PPAR-γ and	
				PPAR-δ mediates metabolic reprogramming regulating CRC.	(88, 89)
				SIRT3 is involved in CRC resistance.	(93)
SIRT4	Mitochondria	418 aa	ADP-ribosyltransferase , Deacetylation	SIRT4 regulates glutamine metabolism through mitochondrial metabolism.	(96, 100)
				CREB2 regulates SIRT4 transcription in a mTORC1 dependent manner.	(96)
				SIRT4 regulates e-cadherin expression and inhibits glutamine metabolism.	(101)
				SIRT4 inhibits CRC through the AKT/GSK-3β/CyclinD1 pathway.	(102)
SIRT5	Mitochondria	SIRT5iso1 (310 aa), SIRT5iso2 (299 aa)	Desuccinylation, Demalonylation, Deglutarylation	SIRT5 inhibits tumor growth by blocking serine catabolism.	(116)
				SIRT5 plays a role in metabolic reprogramming in CRC.	(115)
				SIRT5 interacts with LDHB to regulate CRC.	(117)
SIRT6	Nucleus	Chromosome 19, encoded by 8 and 7 exons.	Deacetylation, ADP-ribosyltransferase	SIRT6 interacts with FoxO3a to initiate SIRT6 transcriptional expression, demonstrating the role of AKT/FoxO3a/SIRT6 axis in promoting CRC.	(131)
				SIRT6 regulates PTEN/AKT, JAK2/STAT3 signaling pathway inhibits cell proliferation, invasion and migration, and promotes apoptosis.	(133, 137)
SIRT7	Nucleus	Chromosome 17q25.3	Deacetylation	SIRT7 promotes the migration, invasion and metastasis of CRC.	(142, 143)
				SIRT7 promotes cell proliferation and metastasis through the MEK/ERK/MAPK, SIRT7/PCAF/MDM2 axis and EMT.	(142, 144)

## 2 SIRT1

### 2.1 SIRT1 in colorectal physiology

SIRT1 is an NAD^+^-dependent deacetylase protein that is mainly monomeric, is composed of 747 amino acids, and is a member of the histone deacetylase (HDAC) III family (31). It is involved mainly in the deacetylation of histone lysines (31), which consume NAD^+^-mediated H3 lysine 9 (H3K9) and indirectly promote the production of trimethyl H3K9 (32). SIRT1 is located mainly in the nucleus, and studies have shown that SIRT1 is transferred from the cytoplasm to the nucleus (23). It is involved in inflammation, apoptosis, cell metabolism, DNA repair and stress resistance through interactions with histones H3 and H4, p53, NF-κB, PPAR-γ coactivator 1α (PGAL-1α) and FOXO family transcription factors (33, 34). SIRT1 in the colorectal mucosa plays an important role in physiological processes.

### 2.2 Behavioural changes in SIRT1 in CRC and potential regulatory mechanisms

SIRT1, a protein whose expression is elevated in cancers such as hepatocellular carcinoma (35), oral squamous cell carcinoma (36), gastric cancer (37) and primary colon cancer (38), plays a multifaceted role in CRC progression. It is implicated in CRC proliferation, migration, invasion and distant metastasis and is correlated with poor patient prognosis (39–44). Mechanistically, SIRT1 drives CRC progression through interactions with miRNAs (e.g., miR-1185-1, miR-141, and miR-15b-5p) and immune evasion pathways. For example, miR-34a deficiency in mice activates the SIRT1/NF-κB/B7-H3/TNF-α axis, facilitating cancer immune evasion (42, 45, 46).

SIRT1 interacts with SAD1/UNC84 domain protein-2 (SUN2), activating the BDNF/TrkB pathway to increase MeCP2 acetylation, which promotes BDNF promoter binding and colon cancer metastasis (47). Additionally, SIRT1 contributes to the malignant transformation of IBD to CRC via p62- or p65-mediated signalling (48, 49). It also activates the Wnt/β-catenin pathway to accelerate CRC progression (50, 51). However, the SIRT1 inhibitor MHY2251 induces CRC apoptosis through the JNK/p53 pathway (52). NAMPT, which is overexpressed in CRC, modulates SIRT1/p53 signalling to induce G0/G1 cell cycle arrest, upregulates p21, and downregulates cyclins (D1, E1, and E2), highlighting a negative regulatory interplay between SIRT1 and p53 (53–56). Metabolically, SIRT1 suppresses glycolysis by relocating deacetylated β-catenin from the nucleus to the cytoplasm while promoting fatty acid oxidation (FAO) (57). Vitamin D enhances SIRT1 activity via K610 deacetylation in colon cancer cells (58). Furthermore, NCAPD2 inhibits autophagy via the Ca²^+^/CAMKK/AMPK/mTORC1 pathway and the PARP-1/SIRT1 axis, driving CRC development (59). Immune regulation studies have revealed that natural killer (NK) and Treg cells modulate SIRT1 expression to mediate CRC immune evasion (34, 60). Overall, SIRT1 orchestrates CRC proliferation, migration, invasion and metastasis through diverse molecular pathways, underscoring its potential as a therapeutic target (**Table 1**; **Figure 1A**).

## 3 SIRT2

### 3.1 The physiological function of SIRT2

SIRT2 is a member of the sirtuin family of genes, located on human chromosome 19, and contains 18 exons. SIRT2 is expressed in 179 species and produces physiologically active transcripts 1 and 2 by different alternative splicing (61, 62). It is a class III HDAC that is predominantly localized to the cytoplasm, and studies have shown that SIRT2 plays important roles in tumour stem cells, ageing, energy metabolism, gene transcription, avoidance of immune destruction, regulation of the cell cycle and cell differentiation (62, 63). SIRT2 is involved in the regulation of intestinal cell proliferation and differentiation, lipid synthesis and fatty acid oxidation and mainly regulates insulin through the PI3K/protein kinase B (PKB/AKT) signalling pathway to maintain blood glucose levels (64–66). SIRT2 plays important roles in multiple life processes.

### 3.2 SIRT2 regulates the biological behaviour of CRC

SIRT2 regulates the tumour microenvironment and liver, gastric and colon cancers (62). According to immunohistochemistry, the expression of SIRT2 in CRC tissues is upregulated relative to that in adjacent tissues, and SIRT2 is located mainly in the cytoplasm of colon epithelial cells (67, 68). Moreover, increased expression of SIRT2 is associated with the clinicopathological characteristics and poor prognosis of patients with colon cancer (67, 69). Silencing SIRT2 inhibits cell proliferation and metastasis *in vitro* (67, 68). Studies have shown that tumour angiogenesis is the key to tumour proliferation and metastasis (69). SIRT2 increases mammalian cell division cycle 42 (CDC42) acetylation, decreases K153 acetylation, and increases the migration and invasion abilities of colon cancer cells (70). Paradoxically, during CRC progression and liver metastasis, SIRT2 deacetylates IDH1 at lysine 224 (K224), significantly suppressing the malignant behaviour of CRC cells both *in vitro* and *in vivo* (71, 72). This highlights the dual functional role of SIRT2 in CRC pathogenesis; while SIRT2 generally promotes CRC malignancy, it can also inhibit malignant behaviours via nonhistone acetylation modifications. SIRT2 is upregulated in CRC and is associated with poor prognosis; however, the mechanism affecting CRC remains unclear and needs further investigation.

### 3.3 The mechanism of SIRT2 in CRC

SIRT2 depends on the STAT3/VEGFA signalling pathway to participate in blood vessel formation. In different CRC cell lines, silencing SIRT2 downregulates STAT3 phosphorylation and inhibits the secretion of VEGFA, weakening the interaction between JAK2 and STAT3 and thereby affecting the STAT3 signalling pathway. In addition, *in vitro* angiogenesis experiments demonstrated that VEGFA stimulation reverses the effect of SIRT2 knockout on CRC angiogenesis (69). In HCT116 cells, the addition of the inhibitor AK-1 downregulates SIRT2 activity and inhibits the NF-κB/CSN2 pathway, and the decreased snail levels lead to the upregulation of p21 (a cyclin-dependent kinase inhibitor), which inhibits the cell G1 phase, inhibiting in proliferation and migration (73). Inhibition of the Wnt/β-catenin signalling pathway increases SIRT2 promoter activity and mRNA and protein expression, whereas activation of the Wnt/β-catenin signalling pathway decreases SIRT2 promoter activity and expression. β-catenin is recruited to the promoter of SIRT2 and transcriptionally regulates SIRT2 expression. Inhibition of Wnt/β-catenin increases mitochondrial oxidative phosphorylation (OXPHOS) and CRC cell differentiation. In addition, inhibition of OXPHOS attenuates Wnt/β-catenin-induced CRC cell differentiation. Similarly, the inhibition or knockout of SIRT2 attenuates Wnt/β-catenin suppression-induced differentiation (68). A study on drug resistance revealed that the absence of SIRT2 activates MEK and ERK to promote CRC cell proliferation, leading to drug resistance via the tyrosine kinase receptor RAS/RAF/MEK/ERK pathway, and impairs the response to the upstream inhibition of EGFR or BRAF (74). Finally, SIRT2 upregulates the antitumour activity of natural killer cells in mice with colorectal mesenteric lymph node failure during immune regulation (75, 76). These results demonstrate that SIRT2 is a novel target gene in colon cancer that is associated with the regulation of multiple signalling pathways, potentially offering new directions for targeted drug therapy in CRC (**Table 1**, **Figure 1B**).

## 4 SIRT3

### 4.1 Tissue and subcellular distribution of SIRT3

SIRT3 is an important mitochondrial protein deacetylase responsible for the deacetylation of serine hydroxymethyl transferase 2 (SHMT2). Containing a conserved enzyme core, two domains responsible for mitochondrial localization at the N-terminus of 25 amino acid residues, SIRT3 activates CREB to stimulate peroxisome proliferator-activated receptor c (PPARc) coactivator-1α(PGC-1α) expression, and PGC-1α activates oestrogen-associated receptor-α (ERRα) to stimulate SIRT3 expression at the mRNA and protein levels (77). SIRT3 expression levels are highest in the kidney, brain, heart, liver, lung, ovary, spleen and thymus (78). In mouse studies, SIRT3 is located in the mitochondria and consists of proteins of 257 amino acids, which is consistent with the COOH-terminal portion of human SIRT3 (residues 143–399) (79, 80). SIRT3 is located mainly in the nucleus and migrates from the nucleus to the mitochondria during cellular stress (81). SIRT3 plays an important role in the metabolic process and inhibits proteins in the anabolic process by deacetylating catabolism, increasing energy storage and maintaining cell energy homeostasis (80). Defects in the SIRT3 regulatory pathway lead to various metabolic disorders and are expected to become new drug targets for the diagnosis and treatment of diseases in the future.

### 4.2 Role of SIRT3 in CRC

SIRT3 is responsible for the deacetylation of SHMT2, which is acetylated at K95 in CRC cells. SHMT2-K95-Ac destroys its functional tetramer structure and inhibits its enzyme activity, and SHMT2-K95-Ac reduces CRC cell proliferation and tumour growth *in vivo* by reducing serine consumption and NADPH levels (82). The SIRT3 expression level is significantly correlated with lymph node metastasis and tumour stage, and high SIRT3 expression is associated with poor prognosis in patients with colon cancer (83, 84). Moreover, biological behaviour experiments have shown that downregulation of the SIRT3 gene inhibits the proliferation, invasion and migration of colon cancer cells and increases cell apoptosis (83, 85). Silencing SIRT3 led to mitochondrial dysfunction and decreased cell viability (86). In addition, SIRT3-mediated deacetylation at K352 reduces the activity of mitochondrial malic enzyme 2 (ME2) and disrupts redox homeostasis, and the ME2 K352R mutation suppresses tumour growth (87). SIRT3 plays important roles in the proliferation, invasion, migration and mucosal barrier of CRC.

### 4.3 SIRT3 regulates the molecular mechanisms of CRC

In autophagy research, δ-valerobetaine (δVB) promotes mitochondrial dysfunction, induces mitochondrial autophagy, and promotes death in SW480 and SW620 colon cancer cells. Mitochondrial dysfunction induced by δVB is related to the dysregulation of SIRT3. δVB downregulates mitochondrial SIRT3 in a time-dependent manner, and autophagy induced by δVB triggers cell apoptosis mediated by the PINK1/Parkin/LC3B axis (88). SIRT3 mediates metabolic reprogramming by regulating PPAR-α, PPAR-γ and PPAR-δ. A recent study revealed that whey-induced growth inhibition and apoptosis were associated with the downregulation of PPAR-α and the positive regulation of PPAR-γ. SIRT3 silencing had the opposite effect on PPAR-γ, PPAR-α and LDHA. These results indicate that SIRT3 directly/indirectly mediates the regulation of mitochondrial dysfunction and metabolism (89). In recent years, SIRT3 has been shown to promote CRC metastasis through JNK-mediated autophagy, and noncoding RNAs have been shown to upregulate H3K27 in the ETS1 promoter to induce SIRT3 expression (90, 91). However, there are few studies on the molecular mechanism by which SIRT3 regulates the occurrence and development of CRC, and further exploration is needed.

### 4.4 SIRT3 is involved in CRC chemotherapy resistance

Recently, a study on the involvement of SIRT3 in CRC cell tolerance revealed that high SIRT3 protein levels were associated with a significant reduction in cancer-specific survival with low SIRT3 protein levels. SIRT3 knockdown increases mtROS levels and cell sensitivity to anticancer drugs. The downregulation of SIRT3 decreases the expression and activity of SOD2, and the downregulation of SOD2 also increases the sensitivity to anticancer drugs (92). In addition, SIRT3 is recruited with PGC-1α under oxidative stress, and SIRT3 downregulation can reduce PGC-1α expression and mitochondrial function. Inhibition of PGC-1α can decrease mitochondrial activity and increase apoptosis in cells treated with antitumour drugs (92). Silencing SIRT3 in colon cancer cells increases ROS levels and ROS production and reduces manganese superoxide dismutase (MnSOD) protein levels and activity, the expression of antioxidant enzyme-encoding genes is significantly reduced, reducing the antioxidant capacity of cells, and oxaliplatin combined with silencing SIRT3 increases ROS production and cell apoptosis (84). Regarding the drug resistance mechanism, MTHFD2 maintains the activity of the MTHFD2 enzyme and the production of NADPH in tumour cells. Moreover, cisplatin inhibits the expression of SIRT3 and upregulates the acetylation level of MTHFD2. Therefore, cisplatin-induced acetylation of MTHFD2 leads to decreases in enzyme activity and NADPH and breaks redox homeostasis in mitochondria (93). These findings indicate that SIRT3 is involved in the progression of CRC through different signalling pathways (**Table 1**, **Figure 2A**).

## 5 SIRT4

### 5.1 Structure and role of SIRT4

SIRT4, a member of the sirtuin family, is located in the mitochondria (94). Mahlknecht et al. isolated and identified the mouse SIRT4 genome sequence, which is encoded by six exons. The 1648 bp mouse Sirt4 transcript encodes a 418-amino-acid protein with a predicted molecular weight of 47.3 kDa (95). SIRT4 lacks nicotinamide adenine dinucleotide-dependent deacetylase activity but has ADP-ribosyltransferase activity on histones (95). SIRT4 plays important roles in cell metabolism, nutrient metabolism, the stress response and longevity (96, 97).

### 5.2 Role of SIRT4 in CRC

*In vivo* and *in vitro* experiments revealed that the overexpression of SIRT4 inhibits the proliferation, cell number and tumour formation of CRC cells; reduces the metabolic capacity of glutamine; and synergizes with glycolysis inhibitors to induce cell death. SIRT4 also increases the sensitivity of CRC cells to the chemotherapy drug 5-fluorouracil (5-FU) by inhibiting the cell cycle (98, 99). Recent studies have shown that SIRT4 acts as a tumour suppressor gene by regulating glutamine metabolism through mitochondrial metabolism (96, 100).

### 5.3 SIRT4 regulates the molecular mechanisms of CRC

In metabolic research, Csibi A et al. identified a new mTORC1 regulatory pathway that controls glutamine-dependent energy generation, mTORC1 regulates glutamine metabolism through GDH and inhibits SIRT4 to control GDH activity, CREB2 regulates SIRT4 transcription in an mTORC1-dependent manner and regulates CREB2 stability (96). mTORC1 activation promotes the binding of CREB2 to βTrCP and regulates CREB2 ubiquitination, whereas SIRT4 inhibits cell proliferation and tumour development (96). These studies revealed the important effects of SIRT4 on glutamine mutation and tumour cell metabolism, providing important clues for SIRT-mediated metabolism. SIRT4 regulation of E-cadherin expression negatively regulates CRC cell progression by inhibiting glutamine metabolism, α-KG eliminates the SIRT4-induced expression of E-cadherin, and SIRT4 inhibits EMT by inactivating GDH and reducing the intracellular α-KG level. In addition, SIRT4 expression decreases with increasing CRC invasion and metastasis. Like E-cadherin, SIRT4 plays a role in the inhibition of the CRC malignant phenotype, which may partly come from its regulation of E-cadherin (101). Reduced SIRT4 expression is associated with a malignant phenotype *in vivo* and *in vitro*, and SIRT4 inhibits the activation of glutaminase, thereby initiating AKT activation. SIRT4-dependent glutaminase inhibits proliferation, migration and invasion through the AKT/GSK3β/CyclinD1 pathway (102). Sodium butyrate inhibits the growth of CRC by inhibiting SIRT4/HIF-1α-mediated aerobic glycolysis (103). However, miR-15a-5p enhances the malignant progression of CRC cells through the STAT3/TWIST1 and PTEN/AKT signalling pathways by targeting SIRT4 (104). In summary, we found that SIRT4 is involved primarily in the metabolic pathways of CRC (**Table 1**, **Figure 2B**).

## 6 SIRT5

### 6.1 The function of SIRT5

SIRT5 is a member of the NAD⁺-dependent sirtuin family and belongs to the class III sirtuin family. The human SIRT5 gene encodes two main subtypes, SIRT5 (iso1) and SIRT5 (iso2) (105), which include posttranslational modifications of desuccinylation, demalonylation and deglutarylation to regulate metabolic enzymes and control the TCA cycle and glycolysis (106, 107); the urea cycle (108); fatty acid oxidation (109, 110); and other metabolic pathways (111, 112). SIRT5 is widely distributed in the brain, heart, liver, kidney, muscle and testicles (113). Some studies have shown that SIRT5 expression is regulated by PGC-1α and AMP-activated protein kinase (AMPK) (114). Therefore, SIRT5 plays a role in maintaining metabolism and cellular homeostasis by regulating various metabolic activities.

### 6.2 SIRT5 is involved in the development of CRC

SIRT5 expression is upregulated in colorectal tissues and is related to tumour size, lymph node metastasis and tumour stage (115). SIRT5 expression is associated with poor prognosis and poor overall survival in patients with CRC (115). Inactivation of SIRT5 leads to downregulation of the SHMT2 enzyme, attenuates SHMT2 desuccinylation, reduces serine catabolism, and thereby suppresses tumour growth (116). Mechanistically, SIRT5 plays a metabolic reprogramming role in CRC. Direct interaction between SIRT5 and glutamate dehydrogenase 1 (GLUD1) leads to deglutarylation and functional activation of GLUD1. K545 in GLUD1 is the main glutarylation target of SIRT5, which is upregulated to activate GLUD1 in a glutarylation-dependent manner to promote glutamine metabolism and is associated with CRC cell proliferation, survival and xenograft tumour growth (115). SIRT5 interacts with LDHB and promotes LDHB deacetylation at the K329 site, increasing LDHB enzyme activity and promoting lysosomal acidification and autophagy, resulting in increased autophagy and reduced apoptotic cell death. Therefore, SIRT5 interacts with LDHB to regulate the autophagy, apoptosis and proliferation of CRC cells (117). In addition, SIRT5 protects CRC from DNA damage and promotes the malignant progression of CRC (118). SIRT5 interacts with citrate synthase (CS), succinylates CS on the evolutionarily conserved residues K393 and K395, and inhibits the proliferation and migration of colon cancer cells (119). SIRT5 promotes the proliferation and metabolism of CRC cells via glutamate metabolism (**Table 1**, **Figure 2C**).

In studies of chemotherapy resistance, SIRT5-positive wild-type Kras CRC cells were resistant to both chemotherapeutic agents and cetuximab. SIRT5 demalonylates and inactivates succinate dehydrogenase complex subunit A (SDHA), leading to the accumulation of the oncometabolite succinate. Succinate binds to and activates the reactive oxygen species-scavenging enzyme thioredoxin reductase 2 (TrxR2), thereby conferring resistance to chemotherapy (120). Another study revealed that 5-FU significantly downregulates the protein expression of SIRT5 and FOXO3a in a p53-independent manner, providing a rationale for combination therapy in CRC (121).

## 7 SIRT6

### 7.1 The function of SIRT6

SIRT6 is a member of the evolutionarily conserved sirtuin family of histone deacetylases with deacetylase and ADP-ribosyltransferase activities. SIRT6 is located on human chromosome 19 and has two isomers encoded by exons 8 and 7 (122, 123). It is expressed mainly in the brain, kidney and heart (122, 124, 125). SIRT6 requires the cellular metabolite NAD^+^ to be involved in telomere stabilization (123), DNA repair and transcriptional regulation and is associated with cell senescence, as well as the transcriptional silencing of segmental telomere and subtelomere regions (123, 126). Moreover, it plays important roles in biological processes such as metabolism, ageing, DNA stabilization and repair, proliferation and differentiation (127–131).

### 7.2 Role of SIRT6 in CRC

SIRT6 is expressed at low levels in colon cancer samples, which is correlated with poor prognosis and reduced overall survival (131, 132). SIRT6 plays a dual role in CRC, and some studies have shown that SIRT6 promotes CRC cell proliferation, suppresses apoptosis, and enhances invasion (132, 133). FoxO3a is associated with SIRT6 promoter activity, mRNA levels and protein expression stimulated by dephosphorylation, which blocks BKM120 (131). However, other studies have reported reduced SIRT6 expression in CRC stem cells (CSCs), where it inhibits the proliferation and colony formation of colorectal CSCs and induces G0/G1 phase arrest. SIRT6 binds to the cell division cycle 25A (CDC25A) promoter and reduces the acetylation level of lysine 9 of histone H3. Therefore, SIRT6 inhibits CRC stem cell proliferation by targeting CDC25A (134). Therefore, SIRT6 plays either protumourigenic or tumour-suppressive roles under different conditions. The selective SIRT6 activator MDL-811 has been shown to activate SIRT6-mediated deacetylation of histone H3 (H3K9Ac, H3K18Ac, H3K56Ac) *in vitro*, with the identification of its downstream target gene cytochrome P450 family 24 subfamily A member 1 (CYP24A1). The combined application of MDL-811 synergistically enhances the anti-CRC effects of vitamin D3, as validated in both *in vitro* and *in vivo* studies (135). SIRT6 inhibits the tumour killing activity of NK cells in CRC mice, and downregulated SIRT6 strongly infiltrates NK cells to inhibit the progression of CRC in mice (136). These results demonstrate that SIRT6 regulates stem cell and tumour immunity in CRC.

### 7.3 SIRT6 participates in the regulation of the CRC signalling pathway

The binding site on the SIRT6 promoter directly interacts with activated FoxO3a, and AKT is inhibited. FoxO3a is dephosphorylated, which results in the translocation of FoxO3a from the cytoplasm to the nucleus, where it binds to the SIRT6 promoter, resulting in the transcription of SIRT6 (131). Therefore, SIRT6 is a new transcriptional target of FoxO3a, and the role of the AKT/FoxO3a/SIRT6 axis in promoting apoptosis and colon cancer treatment with BKM120 *in vitro* and *in vivo* has been demonstrated (131). SIRT6 overexpression inhibits cell proliferation, invasion and migration by regulating the PTEN/AKT signalling pathway and promotes cell apoptosis (137). SIRT6 inhibits colon cancer cell proliferation and induces apoptosis through the JAK2/STAT3 signalling pathway (133). In the study of resistance mechanisms, cisplatin and BKM120 interact to simultaneously express SIRT6 in a p53-dependent and p53-independent manner. Regorafenib induces FoxO3a-dependent SIRT6 expression by inactivating ERK, and the loss of SIRT6 leads to the development of resistance to BKM120 and combination therapy in colon cancer (131). SIRT6 plays dual roles in CRC progression by regulating signalling pathways (**Table 1**, **Figure 3A**).

## 8 SIRT7

### 8.1 The function of SIRT7

SIRT7, a member of the sirtuin family of genes, is located mainly in the nucleus on chromosome 17q25.3 and binds to ribosomal RNA (rRNA) genes to participate in postmitotic rDNA transcription and control rRNA expression (138–140). SIRT7 maintains the oncogenic transformation of human cancer cells by deacetylating the lysine of histone H3 (141). The expression of SIRT7 is upregulated in CRC tissues (142, 143), and in CRC tissues, high expression of SIRT7 is correlated with tumour size; tumour, lymph node, and metastatic stages; and distant metastasis (143). According to functional experiments, SIRT7 promotes the formation, migration, invasion and metastasis of CRC cell colonies (142, 143).

### 8.2 Molecular mechanism of SIRT7 in CRC

Mechanistic research revealed that in CRC cells, SIRT7 overexpression increases MEK1/2 phosphorylation and RAF-1 levels, SIRT7 overexpression promotes liver and lung metastasis of CRC cells, and the addition of the ERK inhibitor PD98059 inhibits SIRT7-induced CRC cell migration. SIRT7 promotes cell proliferation and metastasis through the MEK/ERK/MAPK signalling pathway and EMT (142). GRIM-19 activates SIRT7, triggers PCAF-mediated MDM2 ubiquitination, stabilizes the p53 protein, and enhances the efficacy of oxaliplatin in CRC. Therefore, GRIM-19 inhibits CRC cell proliferation and induces apoptosis in a p53-dependent manner through the SIRT7/PCAF/MDM2 axis (144). In studies on the mechanism of drug resistance, SIRT7 downregulation is mediated by the Tat-binding protein 1 (TBP1) proteasome-dependent pathway. When 5-FU is added to a coculture of CRC cells, TBP1 is dephosphorylated at tyrosine 381, enhancing its interaction with SIRT7. TBP1 targets SIRT7 through ubiquitin-independent and proteasome-dependent pathways, resulting in radiosensitivity and cell death (145). Therefore, SIRT7 is involved in the malignant progression of CRC in multiple ways (**Table 1**, **Figure 3B**).

## 9 Development and application prospects for sirtuin-targeting drugs

In recent years, the development of drugs targeting sirtuins has become a research hotspot, with application prospects spanning cancer therapy, metabolic diseases, neurodegenerative disorders, and antiaging. The novel SIRT1 inhibitor MHY2251 induces apoptosis in CRC cells via the JNK/p53 pathway (52). The sirtuin inhibitor MHY2245 induces cell cycle arrest, triggers apoptosis through caspase activation, causes DNA damage responses, and exerts related anticancer effects (146). Evodiamine (EVO) inhibits the migration and invasion of CRC cells by suppressing SIRT1-mediated acetylation of NF-κB p65 (147). Aspirin exerts anticancer effects by inducing senescence in human CRC cells through the SIRT1–AMPK pathway (148). Novel lysine-based thioureas act as SIRT2 inhibitors with anticancer activity in mouse models of CRC (149). Glycyrrhizic acid exerts potent anticancer activity by inhibiting SIRT3, reducing cell viability, colony formation, invasion, and migration (150). Metformin increases ROS levels and SIRT3 activity to induce cell death and enhance cytotoxicity (151). The combination of SIRT5 inhibitors, chemotherapeutic agents and/or cetuximab represents a critical therapeutic strategy for wild-type Kras CRC (120). Oleanolic acid induces colon cancer cell death through the p38/FOXO3a/SIRT6 pathway (152). The SIRT7 inhibitor YZL-51N induces DNA damage repair in CRC cells and exhibits synergistic anticancer effects when combined with etoposide (153). These studies provide a theoretical foundation for targeting sirtuins in drug development. Notably, sirtuins may play dual tumour-suppressive or oncogenic roles depending on the tissue or disease stage, necessitating the development of tissue-specific or conditionally regulated drugs. The development of sirtuin-targeted drugs has shifted from simple activation/inhibition to precise regulation, integrating metabolic reprogramming, epigenetic modifications, and immune microenvironment modulation, highlighting their broad application potential.

## 10 Conclusions and future perspectives

SIRT1 has dual effects, as it can promote and inhibit cancer. In protumour mechanisms, SIRT1 is highly expressed in CRC and promotes tumour proliferation, metastasis, and chemotherapy resistance by activating the Wnt/β-catenin pathway, regulating miRNAs (e.g., miR-34a deficiency facilitates immune evasion), and suppressing p53 function. Regarding its antitumour potential, SIRT1 inhibits glycolysis via the deacetylation of β-catenin and suppresses tumour metabolism through fatty acid oxidation (FAO). Vitamin D enhances the antitumour effects of SIRT1 by activating its K610 deacetylation. SIRT2 is typically upregulated in CRC tissues, promoting angiogenesis via the STAT3/VEGFA pathway and influencing differentiation through interactions with Wnt/β-catenin. Paradoxically, SIRT2 suppresses CRC liver metastasis by deacetylating IDH1. In chemoresistance, SIRT2 activates TrxR2 via SDHA succinylation to scavenge ROS, leading to multidrug resistance. SIRT3 promotes CRC proliferation by deacetylating SHMT2 to regulate serine metabolism. Knockdown of SIRT3 increases mitochondrial ROS and enhances sensitivity to oxaliplatin. Additionally, SIRT3 modulates metabolic reprogramming via the PPAR pathway, impacting the tumour microenvironment. SIRT5 activates glutamine metabolism by deglutarylating GLUD1, driving CRC cell proliferation. It also enhances lysosomal acidification and autophagy via LDHB deacetylation, inhibiting apoptosis. In wild-type KRAS CRC, SIRT5 accumulates succinate to activate TrxR2, conferring chemotherapy resistance. SIRT6 is generally downregulated in CRC and is linked to poor prognosis but suppresses cancer stem cell (CSC) proliferation. The selective activator MDL-811 enhances vitamin D3’s anti-CRC effects by deacetylating histone H3, with dual roles attributed to AKT/FoxO3a/SIRT6 axis regulation. SIRT7 overexpression is correlated with CRC metastasis, promoting EMT and liver/lung metastasis via the MEK/ERK pathway. GRIM-19 stabilizes p53 through the SIRT7/PCAF/MDM2 axis, improving oxaliplatin efficacy.

Future research directions and therapeutic strategies will involve the development of targeted therapies for sirtuins and inhibitors/activators. Specific agents should be designed based on the pro- or antitumour roles of sirtuins (e.g., SIRT5 inhibitors to block glutamine dependency, and SIRT6 activators such as MDL-811 enhance immunotherapy). Natural compounds such as anthocyanins in berries inhibit CRC progression by activating SIRT6, suggesting the use of polyphenols as adjuvant therapies. Metabolic interventions and combination therapies can target sirtuin-mediated metabolic reprogramming (e.g., glutamine or pentose phosphate pathways). Inhibiting SIRT5 may disrupt nucleotide synthesis and improve chemosensitivity. Immune checkpoint inhibitors (e.g., PD-L1) combined with metabolic modulators (e.g., sulphur amino acid restriction) can have synergistic effects. Regarding epigenetic and microenvironment regulation, the roles of sirtuins in tumour stem cells and immune modulation via histone modifications should be further explored (e.g., SIRT6 suppresses CSCs via CDC25A, and SIRT2 regulates NK cell activity). Sirtuin–gut microbiota interactions should be investigated to develop microbiota–metabolism combination therapies. Personalized treatments and biomarkers can also be used to develop individualized strategies based on sirtuin expression profiles (e.g., high SIRT3 predicts chemoresistance). Dynamic monitoring techniques (e.g., metabolomics or imaging markers) need to be established to track sirtuin activity.

Despite progress, key challenges remain: 1) Functional Complexity: Defining the dual roles of some sirtuins (e.g., SIRT2) across subcellular locations or tumour stages requires further exploration of dynamic regulatory networks. 2) Drug delivery and selectivity: The bioavailability of existing agents (e.g., resveratrol) needs to be improved, and novel small molecules should be developed. 3) Clinical Translation: Advanced multicentre clinical trials should be performed to validate the safety and efficacy of combination therapies. With advancements in metabolomics and single-cell sequencing, the multidimensional regulatory mechanisms of sirtuins in CRC will be further elucidated, paving the way for targeted therapies and precision medicine.

## References

[1] Siegel RL Miller KD Wagle NS Jemal A. Cancer statistics, 2023. CA Cancer J Clin. (2023) 73:17–48. doi: 10.3322/caac.21763, PMID: 36633525

[2] Saputra HA Karim MM. Fundamentals and research progression on electrochemical sensing of colorectal cancer. Mikrochim Acta. (2025) 192:355. doi: 10.1007/s00604-025-07207-9, PMID: 40369291

[3] Chen EX Loree JM Titmuss E Jonker DJ Kennecke HF Berry S. Liver metastases and immune checkpoint inhibitor efficacy in patients with refractory metastatic colorectal cancer: A secondary analysis of a randomised clinical trial. JAMA Netw Open. (2023) 6:e2346094. doi: 10.1001/jamanetworkopen.2023.46094, PMID: 38051531 PMC10698621

[4] Huyghe JR Bien SA Harrison TA Kang HM Chen S Schmit SL. Discovery of common and rare genetic risk variants for colorectal cancer. Nat Genet. (2019) 51:76–87. doi: 10.1038/s41588-018-0286-6, PMID: 30510241 PMC6358437

[5] Schneikert J Behrens J. The canonical wnt signalling pathway and its apc partner in colon cancer development. Gut. (2007) 56:417–25. doi: 10.1136/gut.2006.093310, PMID: 16840506 PMC1856802

[6] Lewis A Segditsas S Deheragoda M Pollard P Jeffery R Nye E. Severe polyposis in apc(1322t) mice is associated with submaximal wnt signalling and increased expression of the stem cell marker lgr5. Gut. (2010) 59:1680–6. doi: 10.1136/gut.2009.193680, PMID: 20926645 PMC3002835

[7] Wang D Liang S Zhang Z Zhao G Hu Y Liang S. A novel pathogenic splice acceptor site germline mutation in intron 14 of the apc gene in a chinese family with familial adenomatous polyposis. Oncotarget. (2017) 8:21327–35. doi: 10.18632/oncotarget.15570, PMID: 28423518 PMC5400587

[8] Mármol I Sánchez-de-Diego C Pradilla Dieste A Cerrada E Rodriguez Yoldi MJ. Colorectal carcinoma: A general overview and future perspectives in colorectal cancer. Int J Mol Sci. (2017) 18:197. doi: 10.3390/ijms18010197, PMID: 28106826 PMC5297828

[9] Carethers JM Jung BH. Genetics and genetic biomarkers in sporadic colorectal cancer. Gastroenterology. (2015) 149:1177–90.e3. doi: 10.1053/j.gastro.2015.06.047, PMID: 26216840 PMC4589489

[10] Vilar E Gruber SB. Microsatellite instability in colorectal cancer-the stable evidence. Nat Rev Clin Oncol. (2010) 7:153–62. doi: 10.1038/nrclinonc.2009.237, PMID: 20142816 PMC3427139

[11] Gelsomino F Barbolini M Spallanzani A Pugliese G Cascinu S. The evolving role of microsatellite instability in colorectal cancer: A review. Cancer Treat Rev. (2016) 51:19–26. doi: 10.1016/j.ctrv.2016.10.005, PMID: 27838401

[12] Fan WX Su F Zhang Y Zhang XL Du YY Gao YJ. Oncological characteristics, treatments and prognostic outcomes in mmr-deficient colorectal cancer. biomark Res. (2024) 12:89. doi: 10.1186/s40364-024-00640-7, PMID: 39183366 PMC11346251

[13] Chubb D Broderick P Frampton M Kinnersley B Sherborne A Penegar S. Genetic diagnosis of high-penetrance susceptibility for colorectal cancer (Crc) is achievable for a high proportion of familial crc by exome sequencing. J Clin Oncol. (2015) 33:426–32. doi: 10.1200/jco.2014.56.5689, PMID: 25559809

[14] Chang J Tian J Yang Y Zhong R Li J Zhai K. A rare missense variant in tcf7l2 associates with colorectal cancer risk by interacting with a gwas-identified regulatory variant in the myc enhancer. Cancer Res. (2018) 78:5164–72. doi: 10.1158/0008-5472.Can-18-0910, PMID: 30026326

[15] Johnson RM Qu X Lin CF Huw LY Venkatanarayan A Sokol E. Arid1a mutations confer intrinsic and acquired resistance to cetuximab treatment in colorectal cancer. Nat Commun. (2022) 13:5478. doi: 10.1038/s41467-022-33172-5, PMID: 36117191 PMC9482920

[16] Li J Pan J Wang L Ji G Dang Y. Colorectal cancer: pathogenesis and targeted therapy. MedComm (2020). (2025) 6:e70127. doi: 10.1002/mco2.70127, PMID: 40060193 PMC11885891

[17] Tan M Peng C Anderson KA Chhoy P Xie Z Dai L. Lysine glutarylation is a protein posttranslational modification regulated by sirt5. Cell Metab. (2014) 19:605–17. doi: 10.1016/j.cmet.2014.03.014, PMID: 24703693 PMC4108075

[18] Carrico C Meyer JG He W Gibson BW Verdin E. The mitochondrial acylome emerges: proteomics, regulation by sirtuins, and metabolic and disease implications. Cell Metab. (2018) 27:497–512. doi: 10.1016/j.cmet.2018.01.016, PMID: 29514063 PMC5863732

[19] Palomer X Aguilar-Recarte D García R Nistal JF Vázquez-Carrera M. Sirtuins: to be or not to be in diabetic cardiomyopathy. Trends Mol Med. (2021) 27:554–71. doi: 10.1016/j.molmed.2021.03.004, PMID: 33839024

[20] Guarente L Franklin H. Epstein lecture: sirtuins, aging, and medicine. N Engl J Med. (2011) 364:2235–44. doi: 10.1056/NEJMra1100831, PMID: 21651395

[21] Morigi M Perico L Benigni A. Sirtuins in renal health and disease. J Am Soc Nephrol. (2018) 29:1799–809. doi: 10.1681/asn.2017111218, PMID: 29712732 PMC6050939

[22] Hershberger KA Martin AS Hirschey MD. Role of nad(+) and mitochondrial sirtuins in cardiac and renal diseases. Nat Rev Nephrol. (2017) 13:213–25. doi: 10.1038/nrneph.2017.5, PMID: 28163307 PMC5508210

[23] Yanagisawa S Baker JR Vuppusetty C Koga T Colley T Fenwick P. The dynamic shuttling of sirt1 between cytoplasm and nuclei in bronchial epithelial cells by single and repeated cigarette smoke exposure. PloS One. (2018) 13:e0193921. doi: 10.1371/journal.pone.0193921, PMID: 29509781 PMC5839577

[24] Serrano L Martínez-Redondo P Marazuela-Duque A Vazquez BN Dooley SJ Voigt P. The tumour suppressor sirt2 regulates cell cycle progression and genome stability by modulating the mitotic deposition of H4k20 methylation. Genes Dev. (2013) 27:639–53. doi: 10.1101/gad.211342.112, PMID: 23468428 PMC3613611

[25] Verdin E Hirschey MD Finley LW Haigis MC. Sirtuin regulation of mitochondria: energy production, apoptosis, and signalling. Trends Biochem Sci. (2010) 35:669–75. doi: 10.1016/j.tibs.2010.07.003, PMID: 20863707 PMC2992946

[26] Haigis MC Mostoslavsky R Haigis KM Fahie K Christodoulou DC Murphy AJ. Sirt4 inhibits glutamate dehydrogenase and opposes the effects of calorie restriction in pancreatic beta cells. Cell. (2006) 126:941–54. doi: 10.1016/j.cell.2006.06.057, PMID: 16959573

[27] Guarente L. Calorie restriction and sirtuins revisited. Genes Dev. (2013) 27:2072–85. doi: 10.1101/gad.227439.113, PMID: 24115767 PMC3850092

[28] Burnett C Valentini S Cabreiro F Goss M Somogyvári M Piper MD. Absence of effects of sir2 overexpression on lifespan in C. Elegans and drosophila. Nature. (2011) 477:482–5. doi: 10.1038/nature10296, PMID: 21938067 PMC3188402

[29] Kanfi Y Naiman S Amir G Peshti V Zinman G Nahum L. The sirtuin sirt6 regulates lifespan in male mice. Nature. (2012) 483:218–21. doi: 10.1038/nature10815, PMID: 22367546

[30] Haigis MC Sinclair DA. Mammalian sirtuins: biological insights and disease relevance. Annu Rev Pathol. (2010) 5:253–95. doi: 10.1146/annurev.pathol.4.110807.092250, PMID: 20078221 PMC2866163

[31] Seto E Yoshida M. Erasers of histone acetylation: the histone deacetylase enzymes. Cold Spring Harb Perspect Biol. (2014) 6:a018713. doi: 10.1101/cshperspect.a018713, PMID: 24691964 PMC3970420

[32] Vaquero A Scher M Lee D Erdjument-Bromage H Tempst P Reinberg D. Human sirt1 interacts with histone H1 and promotes formation of facultative heterochromatin. Mol Cell. (2004) 16:93–105. doi: 10.1016/j.molcel.2004.08.031, PMID: 15469825

[33] Finkel T Deng CX Mostoslavsky R. Recent progress in the biology and physiology of sirtuins. Nature. (2009) 460:587–91. doi: 10.1038/nature08197, PMID: 19641587 PMC3727385

[34] Wang X Song X Fang K Chang X. Cd38 modulates cytokine secretion by nk cells through the sirt1/nf-Kb pathway, suppressing immune surveillance in colorectal cancer. Sci Rep. (2024) 14:28702. doi: 10.1038/s41598-024-79008-8, PMID: 39562615 PMC11577103

[35] Zhang ZY Hong D Nam SH Kim JM Paik YH Joh JW. Sirt1 regulates oncogenesis via a mutant P53-dependent pathway in hepatocellular carcinoma. J Hepatol. (2015) 62:121–30. doi: 10.1016/j.jhep.2014.08.007, PMID: 25131770

[36] Chen IC Chiang WF Huang HH Chen PF Shen YY Chiang HC. Role of sirt1 in regulation of epithelial-to-mesenchymal transition in oral squamous cell carcinoma metastasis. Mol Cancer. (2014) 13:254. doi: 10.1186/1476-4598-13-254, PMID: 25424420 PMC4258025

[37] An Y Wang B Wang X Dong G Jia J Yang Q. Sirt1 inhibits chemoresistance and cancer stemness of gastric cancer by initiating an ampk/foxo3 positive feedback loop. Cell Death Dis. (2020) 11:115. doi: 10.1038/s41419-020-2308-4, PMID: 32051395 PMC7015918

[38] Stünkel W Peh BK Tan YC Nayagam VM Wang X Salto-Tellez M. Function of the sirt1 protein deacetylase in cancer. Biotechnol J. (2007) 2:1360–8. doi: 10.1002/biot.200700087, PMID: 17806102

[39] Yu L Dong L Li H Liu Z Luo Z Duan G. Ubiquitination-mediated degradation of sirt1 by smurf2 suppresses crc cell proliferation and tumorigenesis. Oncogene. (2020) 39:4450–64. doi: 10.1038/s41388-020-1298-0, PMID: 32361710

[40] Wang X Liu S Xu B Liu Y Kong P Li C. Circ-sirt1 promotes colorectal cancer proliferation and emt by recruiting and binding to eif4a3. Anal Cell Pathol (Amst). (2021) 2021:5739769. doi: 10.1155/2021/5739769, PMID: 34660182 PMC8519704

[41] Chen X Sun K Jiao S Cai N Zhao X Zou H. High levels of sirt1 expression enhance tumorigenesis and associate with a poor prognosis of colorectal carcinoma patients. Sci Rep. (2014) 4:7481. doi: 10.1038/srep07481, PMID: 25500546 PMC4265776

[42] Sun LN Zhi Z Chen LY Zhou Q Li XM Gan WJ. Sirt1 suppresses colorectal cancer metastasis by transcriptional repression of mir-15b-5p. Cancer Lett. (2017) 409:104–15. doi: 10.1016/j.canlet.2017.09.001, PMID: 28923398

[43] Yu DF Jiang SJ Pan ZP Cheng WD Zhang WJ Yao XK. Expression and clinical significance of sirt1 in colorectal cancer. Oncol Lett. (2016) 11:1167–72. doi: 10.3892/ol.2015.3982, PMID: 26893713 PMC4738140

[44] Cheng F Su L Yao C Liu L Shen J Liu C. Sirt1 promotes epithelial-mesenchymal transition and metastasis in colorectal cancer by regulating fra-1 expression. Cancer Lett. (2016) 375:274–83. doi: 10.1016/j.canlet.2016.03.010, PMID: 26975631

[45] Wang TW Chern E Hsu CW Tseng KC Chao HM. Sirt1-mediated expression of cd24 and epigenetic suppression of novel tumour suppressor mir-1185–1 increases colorectal cancer stemness. Cancer Res. (2020) 80:5257–69. doi: 10.1158/0008-5472.Can-19-3188, PMID: 33046442

[46] Meng F Yang M Chen Y Chen W Wang W. Mir-34a induces immunosuppression in colorectal carcinoma through modulating a sirt1/nf-Kb/B7-H3/tnf-A Axis. Cancer Immunol Immunother. (2021) 70:2247–59. doi: 10.1007/s00262-021-02862-2, PMID: 33492448 PMC10991903

[47] Liu L Li SW Yuan W Tang J Sang Y. Downregulation of sun2 promotes metastasis of colon cancer by activating bdnf/trkb signalling by interacting with sirt1. J Pathol. (2021) 254:531–42. doi: 10.1002/path.5697, PMID: 33931868

[48] Jiang R Fang Z Lai Y Li L Tan J Yu C. Sophocarpine alleviates intestinal fibrosis via inhibition of inflammation and fibroblast into myofibroblast transition by targeting the sirt1/P65 signalling axis. Eur J Pharmacol. (2024) 967:176318. doi: 10.1016/j.ejphar.2024.176318, PMID: 38309678

[49] Chen J Li G He X Chen X Chen Z Liu D. Elmo1 ameliorates intestinal epithelial cellular senescence via sirt1/P65 signalling in inflammatory bowel disease-related fibrosis. Gastroenterol Rep (Oxf). (2024) 12:goae045. doi: 10.1093/gastro/goae045, PMID: 38756351 PMC11096966

[50] Zhang Q Fei S Zhao Y Liu S Wu X Lu L. Pus7 promotes the proliferation of colorectal cancer cells by directly stabilizing sirt1 to activate the wnt/B-catenin pathway. Mol Carcinog. (2023) 62:160–73. doi: 10.1002/mc.23473, PMID: 36222184

[51] García-Martínez JM Chocarro-Calvo A Martínez-Useros J Regueira-Acebedo N Fernández-Aceñero MJ Muñoz A. Sirt1 mediates the antagonism of wnt/B-catenin pathway by vitamin D in colon carcinoma cells. Int J Biol Sci. (2024) 20:5495–509. doi: 10.7150/ijbs.95875, PMID: 39494323 PMC11528448

[52] Kang YJ Kwon YH Jang JY Lee JH Lee S Park Y. Mhy2251, a new sirt1 inhibitor, induces apoptosis via jnk/P53 pathway in hct116 human colorectal cancer cells. Biomol Ther (Seoul). (2023) 31:73–81. doi: 10.4062/biomolther.2022.044, PMID: 35811306 PMC9810441

[53] Pan JH Zhou H Zhu SB Huang JL Zhao XX Ding H. Nicotinamide phosphoribosyl transferase regulates cell growth via the sirt1/P53 signalling pathway and is a prognosis marker in colorectal cancer. J Cell Physiol. (2019) 234:4385–95. doi: 10.1002/jcp.27228, PMID: 30191976

[54] Brockmueller A Buhrmann C Shayan P Shakibaei M. Resveratrol induces apoptosis by modulating the reciprocal crosstalk between P53 and sirt-1 in the crc tumour microenvironment. Front Immunol. (2023) 14:1225530. doi: 10.3389/fimmu.2023.1225530, PMID: 37575245 PMC10413256

[55] Wang XW Jiang YH Ye W Shao CF Xie JJ Li X. Sirt1 promotes the progression and chemoresistance of colorectal cancer through the P53/mir-101/kpna3 axis. Cancer Biol Ther. (2023) 24:2235770. doi: 10.1080/15384047.2023.2235770, PMID: 37575080 PMC10431729

[56] Jia X Liu H Ren X Li P Song R Li X. Nucleolar protein noc4l inhibits tumorigenesis and progression by attenuating sirt1-mediated P53 deacetylation. Oncogene. (2022) 41:4474–84. doi: 10.1038/s41388-022-02447-y, PMID: 36030331

[57] Wei Z Xia J Li J Cai J Shan J Zhang C. Sirt1 promotes glucolipid metabolic conversion to facilitate tumour development in colorectal carcinoma. Int J Biol Sci. (2023) 19:1925–40. doi: 10.7150/ijbs.76704, PMID: 37063423 PMC10092765

[58] García-Martínez JM Chocarro-Calvo A Martínez-Useros J Fernández-Aceñero MJ Fiuza MC Cáceres-Rentero J. Vitamin D induces sirt1 activation through K610 deacetylation in colon cancer. Elife. (2023) 12:RP86913. doi: 10.7554/eLife.86913, PMID: 37530744 PMC10396337

[59] Jing Z He X Jia Z Sa Y Yang B Liu P. Ncapd2 inhibits autophagy by regulating ca(2+)/camkk2/ampk/mtorc1 pathway and parp-1/sirt1 axis to promote colorectal cancer. Cancer Lett. (2021) 520:26–37. doi: 10.1016/j.canlet.2021.06.029, PMID: 34229059

[60] Zi R Zhao X Liu L Wang Y Zhang R Bian Z. Metabolic-immune suppression mediated by the sirt1-cx3cl1 axis induces functional enhancement of regulatory T cells in colorectal carcinoma. Adv Sci (Weinh). (2025) 12:e2404734. doi: 10.1002/advs.202404734, PMID: 39783838 PMC12061293

[61] Rack JG VanLinden MR Lutter T Aasland R Ziegler M. Constitutive nuclear localization of an alternatively spliced sirtuin-2 isoform. J Mol Biol. (2014) 426:1677–91. doi: 10.1016/j.jmb.2013.10.027, PMID: 24177535

[62] Chen G Huang P Hu C. The role of sirt2 in cancer: A novel therapeutic target. Int J Cancer. (2020) 147:3297–304. doi: 10.1002/ijc.33118, PMID: 32449165

[63] O’Callaghan C Vassilopoulos A. Sirtuins at the crossroads of stemness, aging, and cancer. Aging Cell. (2017) 16:1208–18. doi: 10.1111/acel.12685, PMID: 28994177 PMC5676072

[64] Li C Zhou Y Rychahou P Weiss HL Lee EY Perry CL. Sirt2 contributes to the regulation of intestinal cell proliferation and differentiation. Cell Mol Gastroenterol Hepatol. (2020) 10:43–57. doi: 10.1016/j.jcmgh.2020.01.004, PMID: 31954883 PMC7210478

[65] Pereira JM Chevalier C Chaze T Gianetto Q Impens F Matondo M. Infection reveals a modification of sirt2 critical for chromatin association. Cell Rep. (2018) 23:1124–37. doi: 10.1016/j.celrep.2018.03.116, PMID: 29694890 PMC5946459

[66] Ramakrishnan G Davaakhuu G Kaplun L Chung WC Rana A Atfi A. Sirt2 deacetylase is a novel akt binding partner critical for akt activation by insulin. J Biol Chem. (2014) 289:6054–66. doi: 10.1074/jbc.M113.537266, PMID: 24446434 PMC3937672

[67] Zhang LL Zhan L Jin YD Min ZL Wei C Wang Q. Sirt2 mediated antitumor effects of shikonin on metastatic colorectal cancer. Eur J Pharmacol. (2017) 797:1–8. doi: 10.1016/j.ejphar.2017.01.008, PMID: 28088387

[68] Li C Zhou Y Kim JT Sengoku T Alstott MC Weiss HL. Regulation of sirt2 by wnt/B-catenin signalling pathway in colorectal cancer cells. Biochim Biophys Acta Mol Cell Res. (2021) 1868:118966. doi: 10.1016/j.bbamcr.2021.118966, PMID: 33450304 PMC7939112

[69] Hu F Sun X Li G Wu Q Chen Y Yang X. Inhibition of sirt2 limits tumour angiogenesis via inactivation of the stat3/vegfa signalling pathway. Cell Death Dis. (2018) 10:9. doi: 10.1038/s41419-018-1260-z, PMID: 30584257 PMC6315023

[70] Wang DN Ni JJ Li JH Gao YQ Ni FJ Zhang ZZ. Bacterial infection promotes tumorigenesis of colorectal cancer via regulating cdc42 acetylation. PloS Pathog. (2023) 19:e1011189. doi: 10.1371/journal.ppat.1011189, PMID: 36812247 PMC9987831

[71] Wang B Ye Y Yang X Liu B Wang Z Chen S. Sirt2-dependent idh1 deacetylation inhibits colorectal cancer and liver metastases. EMBO Rep. (2020) 21:e48183. doi: 10.15252/embr.201948183, PMID: 32141187 PMC7132198

[72] Wang B Zhao L Yang C Lin Y Wang S Ye Y. Idh1 K224 acetylation promotes colorectal cancer via mir-9-5p/nhe1 axis-mediated regulation of acidic microenvironment. iScience. (2023) 26:107206. doi: 10.1016/j.isci.2023.107206, PMID: 37456829 PMC10339209

[73] Cheon MG Kim W Choi M Kim JE. Ak-1, a specific sirt2 inhibitor, induces cell cycle arrest by downregulating snail in hct116 human colon carcinoma cells. Cancer Lett. (2015) 356:637–45. doi: 10.1016/j.canlet.2014.10.012, PMID: 25312940

[74] Bajpe PK Prahallad A Horlings H Nagtegaal I Beijersbergen R Bernards R. A chromatin modifier genetic screen identifies sirt2 as a modulator of response to targeted therapies through the regulation of mek kinase activity. Oncogene. (2015) 34:531–6. doi: 10.1038/onc.2013.588, PMID: 24469059

[75] Jiang B Ke C Zhou H Xia T Xie X Xu H. Sirtuin 2 up-regulation suppresses the anti-tumour activity of exhausted natural killer cells in mesenteric lymph nodes in murine colorectal carcinoma. Scand J Immunol. (2023) 98:e13317. doi: 10.1111/sji.13317, PMID: 38441393

[76] Ge L Xu M Huang M Liu S Zhou Z Xia Z. Sirtuin2 Suppresses the Polarization of Regulatory T Cells toward T Helper 17 Cells through Repressing the Expression of Signal Transducer and Activator of Transcription 3 in a Mouse Colitis Model. Immun Inflammation Dis. (2024) 12:e1160. doi: 10.1002/iid3.1160, PMID: 38415949 PMC10836035

[77] Chen Y Fu LL Wen X Wang XY Liu J Cheng Y. Sirtuin-3 (Sirt3), a therapeutic target with oncogenic and tumour-suppressive function in cancer. Cell Death Dis. (2014) 5:e1047. doi: 10.1038/cddis.2014.14, PMID: 24503539 PMC3944233

[78] Jin L Galonek H Israelian K Choy W Morrison M Xia Y. Biochemical characterization, localization, and tissue distribution of the longer form of mouse sirt3. Protein Sci. (2009) 18:514–25. doi: 10.1002/pro.50, PMID: 19241369 PMC2760358

[79] Yang YH Chen YH Zhang CY Nimmakayalu MA Ward DC Weissman S. Cloning and characterization of two mouse genes with homology to the yeast sir2 gene. Genomics. (2000) 69:355–69. doi: 10.1006/geno.2000.6360, PMID: 11056054

[80] Nogueiras R Habegger KM Chaudhary N Finan B Banks AS Dietrich MO. Sirtuin 1 and sirtuin 3: physiological modulators of metabolism. Physiol Rev. (2012) 92:1479–514. doi: 10.1152/physrev.00022.2011, PMID: 22811431 PMC3746174

[81] Scher MB Vaquero A Reinberg D. Sirt3 is a nuclear nad+-dependent histone deacetylase that translocates to the mitochondria upon cellular stress. Genes Dev. (2007) 21:920–8. doi: 10.1101/gad.1527307, PMID: 17437997 PMC1847710

[82] Wei Z Song J Wang G Cui X Zheng J Tang Y. Deacetylation of serine hydroxymethyl-transferase 2 by sirt3 promotes colorectal carcinogenesis. Nat Commun. (2018) 9:4468. doi: 10.1038/s41467-018-06812-y, PMID: 30367038 PMC6203763

[83] Liu C Huang Z Jiang H Shi F. The sirtuin 3 expression profile is associated with pathological and clinical outcomes in colon cancer patients. BioMed Res Int. (2014) 2014:871263. doi: 10.1155/2014/871263, PMID: 25105144 PMC4101237

[84] Torrens-Mas M Hernández-López R Oliver J Roca P Sastre-Serra J. Sirtuin 3 silencing improves oxaliplatin efficacy through acetylation of mnsod in colon cancer. J Cell Physiol. (2018) 233:6067–76. doi: 10.1002/jcp.26443, PMID: 29323702

[85] Gan L Li Q Nie W Zhang Y Jiang H Tan C. Prox1-mediated epigenetic silencing of sirt3 contributes to proliferation and glucose metabolism in colorectal cancer. Int J Biol Sci. (2023) 19:50–65. doi: 10.7150/ijbs.73530, PMID: 36594098 PMC9760442

[86] Torrens-Mas M Hernández-López R Pons DG Roca P Oliver J Sastre-Serra J. Sirtuin 3 silencing impairs mitochondrial biogenesis and metabolism in colon cancer cells. Am J Physiol Cell Physiol. (2019) 317:C398–c404. doi: 10.1152/ajpcell.00112.2019, PMID: 31188638

[87] Li C Ge C Wang Q Teng P Jia H Yao S. Sirtuin 3-mediated delactylation of Malic enzyme 2 disrupts redox balance and inhibits colorectal cancer growth. Cell Oncol (Dordr). (2025) 48:979–90. doi: 10.1007/s13402-025-01058-5, PMID: 40192942 PMC12238175

[88] D’Onofrio N Martino E Mele L Colloca A Maione M Cautela D. Colorectal cancer apoptosis induced by dietary Δ-valerobetaine involves pink1/parkin dependent-mitophagy and sirt3. Int J Mol Sci. (2021) 22:8117. doi: 10.3390/ijms22158117, PMID: 34360883 PMC8348679

[89] D’Onofrio N Martino E Balestrieri A Mele L Neglia G Balestrieri ML. Sirt3 and metabolic reprogramming mediate the antiproliferative effects of whey in human colon cancer cells. Cancers (Basel). (2021) 13:5196. doi: 10.3390/cancers13205196, PMID: 34680344 PMC8533739

[90] Liao M Sun X Zheng W Wu M Wang Y Yao J. Linc00922 Decoys Sirt3 to Facilitate the Metastasis of Colorectal Cancer through up-Regulation the H3k27 Crotonylation of Ets1 Promoter. Mol Cancer. (2023) 22:163. doi: 10.1186/s12943-023-01859-y, PMID: 37789393 PMC10548613

[91] Abdelmaksoud NM Abulsoud AI Abdelghany TM Elshaer SS Samaha A Maurice NW. Targeting the sirt3/mnsod and jnk/hmgb1/beclin 1 axes: role of apigenin in multifaceted metabolic intervention in colorectal cancer. J Biochem Mol Toxicol. (2025) 39:e70095. doi: 10.1002/jbt.70095, PMID: 39692359

[92] Paku M Haraguchi N Takeda M Fujino S Ogino T Takahashi H. Sirt3-mediated sod2 and pgc-1α Contribute to chemoresistance in colorectal cancer cells. Ann Surg Oncol. (2021) 28:4720–32. doi: 10.1245/s10434-020-09373-x, PMID: 33393034

[93] Wan X Wang C Huang Z Zhou D Xiang S Qi Q. Cisplatin inhibits sirt3-deacetylation mthfd2 to disturb cellular redox balance in colorectal cancer cell. Cell Death Dis. (2020) 11:649. doi: 10.1038/s41419-020-02825-y, PMID: 32811824 PMC7434776

[94] Laurent G German NJ Saha AK de Boer VC Davies M Koves TR. Sirt4 coordinates the balance between lipid synthesis and catabolism by repressing malonyl coa decarboxylase. Mol Cell. (2013) 50:686–98. doi: 10.1016/j.molcel.2013.05.012, PMID: 23746352 PMC3721068

[95] Mahlknecht U Voelter-Mahlknecht S. Fluorescence in situ hybridization and chromosomal organization of the sirtuin 4 gene (Sirt4) in the mouse. Biochem Biophys Res Commun. (2009) 382:685–90. doi: 10.1016/j.bbrc.2009.03.092, PMID: 19306844

[96] Csibi A Fendt SM Li C Poulogiannis G Choo AY Chapski DJ. The mtorc1 pathway stimulates glutamine metabolism and cell proliferation by repressing sirt4. Cell. (2013) 153:840–54. doi: 10.1016/j.cell.2013.04.023, PMID: 23663782 PMC3684628

[97] Mathias RA Greco TM Oberstein A Budayeva HG Chakrabarti R Rowland EA. Sirtuin 4 is a lipoamidase regulating pyruvate dehydrogenase complex activity. Cell. (2014) 159:1615–25. doi: 10.1016/j.cell.2014.11.046, PMID: 25525879 PMC4344121

[98] Huang G Cheng J Yu F Liu X Yuan C Liu C. Clinical and therapeutic significance of sirtuin-4 expression in colorectal cancer. Oncol Rep. (2016) 35:2801–10. doi: 10.3892/or.2016.4685, PMID: 26986234

[99] Zhu Y Wang G Li X Wang T Weng M Zhang Y. Knockout of sirt4 decreases chemosensitivity to 5-fu in colorectal cancer cells. Oncol Lett. (2018) 16:1675–81. doi: 10.3892/ol.2018.8850, PMID: 30008852 PMC6036483

[100] Jeong SM Xiao C Finley LW Lahusen T Souza AL Pierce K. Sirt4 has tumour-suppressive activity and regulates the cellular metabolic response to DNA damage by inhibiting mitochondrial glutamine metabolism. Cancer Cell. (2013) 23:450–63. doi: 10.1016/j.ccr.2013.02.024, PMID: 23562301 PMC3650305

[101] Miyo M Yamamoto H Konno M Colvin H Nishida N Koseki J. Tumour-suppressive function of sirt4 in human colorectal cancer. Br J Cancer. (2015) 113:492–9. doi: 10.1038/bjc.2015.226, PMID: 26086877 PMC4522635

[102] Cui Y Bai Y Yang J Yao Y Zhang C Liu C. Sirt4 is the molecular switch mediating cellular proliferation in colorectal cancer through gls mediated activation of akt/gsk3β/cyclind1 pathway. Carcinogenesis. (2021) 42:481–92. doi: 10.1093/carcin/bgaa134, PMID: 33315089

[103] Zhang Q Qin Y Sun X Bian Z Liu L Liu H. Sodium butyrate blocks the growth of colorectal cancer by inhibiting the aerobic glycolysis mediated by sirt4/hif-1α. Chem Biol Interact. (2024) 403:111227. doi: 10.1016/j.cbi.2024.111227, PMID: 39241941

[104] Deng J Wang H Liang Y Zhao L Li Y Yan Y. Mir-15a-5p enhances the Malignant phenotypes of colorectal cancer cells through the stat3/twist1 and pten/akt signaling pathways by targeting sirt4. Cell Signal. (2023) 101:110517. doi: 10.1016/j.cellsig.2022.110517, PMID: 36332797

[105] Matsushita N Yonashiro R Ogata Y Sugiura A Nagashima S Fukuda T. Distinct regulation of mitochondrial localization and stability of two human sirt5 isoforms. Genes Cells. (2011) 16:190–202. doi: 10.1111/j.1365-2443.2010.01475.x, PMID: 21143562

[106] Nishida Y Rardin MJ Carrico C He W Sahu AK Gut P. Sirt5 regulates both cytosolic and mitochondrial protein malonylation with glycolysis as a major target. Mol Cell. (2015) 59:321–32. doi: 10.1016/j.molcel.2015.05.022, PMID: 26073543 PMC4571487

[107] Park J Chen Y Tishkoff DX Peng C Tan M Dai L. Sirt5-mediated lysine desuccinylation impacts diverse metabolic pathways. Mol Cell. (2013) 50:919–30. doi: 10.1016/j.molcel.2013.06.001, PMID: 23806337 PMC3769971

[108] Nakagawa T Lomb DJ Haigis MC Guarente L. Sirt5 deacetylates carbamoyl phosphate synthetase 1 and regulates the urea cycle. Cell. (2009) 137:560–70. doi: 10.1016/j.cell.2009.02.026, PMID: 19410549 PMC2698666

[109] Rardin MJ He W Nishida Y Newman JC Carrico C Danielson SR. Sirt5 regulates the mitochondrial lysine succinylome and metabolic networks. Cell Metab. (2013) 18:920–33. doi: 10.1016/j.cmet.2013.11.013, PMID: 24315375 PMC4105152

[110] Sadhukhan S Liu X Ryu D Nelson OD Stupinski JA Li Z. Metabolomics-assisted proteomics identifies succinylation and sirt5 as important regulators of cardiac function. Proc Natl Acad Sci U.S.A. (2016) 113:4320–5. doi: 10.1073/pnas.1519858113, PMID: 27051063 PMC4843474

[111] Di Emidio G Falone S Artini PG Amicarelli F D’Alessandro AM Tatone C. Mitochondrial sirtuins in reproduction. Antioxidants (Basel). (2021) 10:1047. doi: 10.3390/antiox10071047, PMID: 34209765 PMC8300669

[112] Baur JA Chen D Chini EN Chua K Cohen HY de Cabo R. Dietary restriction: standing up for sirtuins. Science. (2010) 329:1012–3; author reply 3-4. doi: 10.1126/science.329.5995.1012, PMID: 20798296 PMC3985480

[113] Michishita E Park JY Burneskis JM Barrett JC Horikawa I. Evolutionarily conserved and nonconserved cellular localizations and functions of human sirt proteins. Mol Biol Cell. (2005) 16:4623–35. doi: 10.1091/mbc.e05-01-0033, PMID: 16079181 PMC1237069

[114] Buler M Aatsinki SM Izzi V Uusimaa J Hakkola J. Sirt5 is under the control of pgc-1α and ampk and is involved in regulation of mitochondrial energy metabolism. FASEB J. (2014) 28:3225–37. doi: 10.1096/fj.13-245241, PMID: 24687991

[115] Wang YQ Wang HL Xu J Tan J Fu LN Wang JL. Sirtuin5 contributes to colorectal carcinogenesis by enhancing glutaminolysis in a deglutarylation-dependent manner. Nat Commun. (2018) 9:545. doi: 10.1038/s41467-018-02951-4, PMID: 29416026 PMC5803207

[116] Yang X Wang Z Li X Liu B Liu M Liu L. Shmt2 desuccinylation by sirt5 drives cancer cell proliferation. Cancer Res. (2018) 78:372–86. doi: 10.1158/0008-5472.Can-17-1912, PMID: 29180469

[117] Shi L Yan H An S Shen M Jia W Zhang R. Sirt5-mediated deacetylation of ldhb promotes autophagy and tumorigenesis in colorectal cancer. Mol Oncol. (2019) 13:358–75. doi: 10.1002/1878-0261.12408, PMID: 30443978 PMC6360364

[118] Wang HL Chen Y Wang YQ Tao EW Tan J Liu QQ. Sirtuin5 protects colorectal cancer from DNA damage by keeping nucleotide availability. Nat Commun. (2022) 13:6121. doi: 10.1038/s41467-022-33903-8, PMID: 36253417 PMC9576705

[119] Ren M Yang X Bie J Wang Z Liu M Li Y. Citrate synthase desuccinylation by sirt5 promotes colon cancer cell proliferation and migration. Biol Chem. (2020) 401:1031–9. doi: 10.1515/hsz-2020-0118, PMID: 32284438

[120] Du Z Liu X Chen T Gao W Wu Z Hu Z. Targeting a sirt5-positive subpopulation overcomes multidrug resistance in wild-type kras colorectal carcinomas. Cell Rep. (2018) 22:2677–89. doi: 10.1016/j.celrep.2018.02.037, PMID: 29514096

[121] Ekremoglu O Koc A. The role of sirt5 and P53 proteins in the sensitivity of colon cancer cells to chemotherapeutic agent 5-fluorouracil. Mol Biol Rep. (2021) 48:5485–95. doi: 10.1007/s11033-021-06558-9, PMID: 34279763

[122] Huang Z Zhao J Deng W Chen Y Shang J Song K. Identification of a cellularly active sirt6 allosteric activator. Nat Chem Biol. (2018) 14:1118–26. doi: 10.1038/s41589-018-0150-0, PMID: 30374165

[123] Michishita E McCord RA Berber E Kioi M Padilla-Nash H Damian M. Sirt6 is a histone H3 lysine 9 deacetylase that modulates telomeric chromatin. Nature. (2008) 452:492–6. doi: 10.1038/nature06736, PMID: 18337721 PMC2646112

[124] Zhang W Wan H Feng G Qu J Wang J Jing Y. Sirt6 deficiency results in developmental retardation in cynomolgus monkeys. Nature. (2018) 560:661–5. doi: 10.1038/s41586-018-0437-z, PMID: 30135584

[125] Saiyang X Deng W Qizhu T. Sirtuin 6: A potential therapeutic target for cardiovascular diseases. Pharmacol Res. (2021) 163:105214. doi: 10.1016/j.phrs.2020.105214, PMID: 33007414

[126] Tennen RI Bua DJ Wright WE Chua KF. Sirt6 is required for maintenance of telomere position effect in human cells. Nat Commun. (2011) 2:433. doi: 10.1038/ncomms1443, PMID: 21847107 PMC3528101

[127] Zhong L D’Urso A Toiber D Sebastian C Henry RE Vadysirisack DD. The histone deacetylase sirt6 regulates glucose homeostasis via hif1alpha. Cell. (2010) 140:280–93. doi: 10.1016/j.cell.2009.12.041, PMID: 20141841 PMC2821045

[128] Sebastián C Zwaans BM Silberman DM Gymrek M Goren A Zhong L. The histone deacetylase sirt6 is a tumour suppressor that controls cancer metabolism. Cell. (2012) 151:1185–99. doi: 10.1016/j.cell.2012.10.047, PMID: 23217706 PMC3526953

[129] Masri S Rigor P Cervantes M Ceglia N Sebastian C Xiao C. Partitioning circadian transcription by sirt6 leads to segregated control of cellular metabolism. Cell. (2014) 158:659–72. doi: 10.1016/j.cell.2014.06.050, PMID: 25083875 PMC5472354

[130] Lyssiotis CA Cantley LC. Sirt6 puts cancer metabolism in the driver’s seat. Cell. (2012) 151:1155–6. doi: 10.1016/j.cell.2012.11.020, PMID: 23217699 PMC3757516

[131] Zhang Y Nie L Xu K Fu Y Zhong J Gu K. Sirt6, a novel direct transcriptional target of foxo3a, mediates colon cancer therapy. Theranostics. (2019) 9:2380–94. doi: 10.7150/thno.29724, PMID: 31149050 PMC6531295

[132] Geng CH Zhang CL Zhang JY Gao P He M Li YL. Overexpression of sirt6 is a novel biomarker of Malignant human colon carcinoma. J Cell Biochem. (2018) 119:3957–67. doi: 10.1002/jcb.26539, PMID: 29227545

[133] Li N Mao D Cao Y Li H Ren F Li K. Downregulation of sirt6 by mir-34c-5p is associated with poor prognosis and promotes colon cancer proliferation through inhibiting apoptosis via the jak2/stat3 signaling pathway. Int J Oncol. (2018) 52:1515–27. doi: 10.3892/ijo.2018.4304, PMID: 29512698 PMC5873872

[134] Liu W Wu M Du H Shi X Zhang T Li J. Sirt6 inhibits colorectal cancer stem cell proliferation by targeting cdc25a. Oncol Lett. (2018) 15:5368–74. doi: 10.3892/ol.2018.7989, PMID: 29552180 PMC5840749

[135] Shang J Zhu Z Chen Y Song J Huang Y Song K. Small-molecule activating sirt6 elicits therapeutic effects and synergistically promotes anti-tumour activity of vitamin D(3) in colorectal cancer. Theranostics. (2020) 10:5845–64. doi: 10.7150/thno.44043, PMID: 32483423 PMC7255010

[136] Xiao F Hu B Si Z Yang H Xie J. Sirtuin 6 is a negative regulator of the anti-tumour function of natural killer cells in murine inflammatory colorectal cancer. Mol Immunol. (2023) 158:68–78. doi: 10.1016/j.molimm.2023.04.011, PMID: 37146480

[137] Tian J Yuan L. Sirtuin 6 inhibits colon cancer progression by modulating pten/akt signaling. BioMed Pharmacother. (2018) 106:109–16. doi: 10.1016/j.biopha.2018.06.070, PMID: 29957460

[138] Grob A Roussel P Wright JE McStay B Hernandez-Verdun D Sirri V. Involvement of sirt7 in resumption of rdna transcription at the exit from mitosis. J Cell Sci. (2009) 122:489–98. doi: 10.1242/jcs.042382, PMID: 19174463 PMC2714433

[139] Ford E Voit R Liszt G Magin C Grummt I Guarente L. Mammalian sir2 homolog sirt7 is an activator of rna polymerase I transcription. Genes Dev. (2006) 20:1075–80. doi: 10.1101/gad.1399706, PMID: 16618798 PMC1472467

[140] Voelter-Mahlknecht S Letzel S Mahlknecht U. Fluorescence in situ hybridization and chromosomal organization of the human sirtuin 7 gene. Int J Oncol. (2006) 28:899–908. doi: 10.3892/ijo.28.4.899 16525639

[141] Barber MF Michishita-Kioi E Xi Y Tasselli L Kioi M Moqtaderi Z. Sirt7 links H3k18 deacetylation to maintenance of oncogenic transformation. Nature. (2012) 487:114–8. doi: 10.1038/nature11043, PMID: 22722849 PMC3412143

[142] Yu H Ye W Wu J Meng X Liu RY Ying X. Overexpression of sirt7 exhibits oncogenic property and serves as a prognostic factor in colorectal cancer. Clin Cancer Res. (2014) 20:3434–45. doi: 10.1158/1078-0432.Ccr-13-2952, PMID: 24771643

[143] Deng Z Wang X Long X Liu W Xiang C Bao F. Sirtuin 7 promotes colorectal carcinoma proliferation and invasion through the inhibition of E-cadherin. Exp Ther Med. (2018) 15:2333–42. doi: 10.3892/etm.2017.5673, PMID: 29467843 PMC5792759

[144] Wang D Wei X Chen X Wang Q Zhang J Kalvakolanu DV. Grim-19 inhibits proliferation and induces apoptosis in a P53-dependent manner in colorectal cancer cells through the sirt7/pcaf/mdm2 axis. Exp Cell Res. (2021) 407:112799. doi: 10.1016/j.yexcr.2021.112799, PMID: 34461110

[145] Tang M Lu X Zhang C Du C Cao L Hou T. Downregulation of sirt7 by 5-fluorouracil induces radiosensitivity in human colorectal cancer. Theranostics. (2017) 7:1346–59. doi: 10.7150/thno.18804, PMID: 28435470 PMC5399598

[146] Kang YJ Jang JY Kwon YH Lee JH Lee S Park Y. Mhy2245, a sirtuin inhibitor, induces cell cycle arrest and apoptosis in hct116 human colorectal cancer cells. Int J Mol Sci. (2022) 23:1590. doi: 10.3390/ijms23031590, PMID: 35163511 PMC8835956

[147] Zhou P Li XP Jiang R Chen Y Lv XT Guo XX. Evodiamine inhibits migration and invasion by sirt1-mediated post-translational modulations in colorectal cancer. Anticancer Drugs. (2019) 30:611–7. doi: 10.1097/cad.0000000000000760, PMID: 30789361 PMC6530977

[148] Jung YR Kim EJ Choi HJ Park JJ Kim HS Lee YJ. Aspirin targets sirt1 and ampk to induce senescence of colorectal carcinoma cells. Mol Pharmacol. (2015) 88:708–19. doi: 10.1124/mol.115.098616, PMID: 26219912

[149] Farooqi AS Hong JY Cao J Lu X Price IR Zhao Q. Novel lysine-based thioureas as mechanism-based inhibitors of sirtuin 2 (Sirt2) with anticancer activity in a colorectal cancer murine model. J Med Chem. (2019) 62:4131–41. doi: 10.1021/acs.jmedchem.9b00191, PMID: 30986062 PMC6901289

[150] Zuo Z He L Duan X Peng Z Han J. Glycyrrhizic acid exhibits strong anticancer activity in colorectal cancer cells via sirt3 inhibition. Bioengineered. (2022) 13:2720–31. doi: 10.1080/21655979.2021.2001925, PMID: 34747319 PMC8974138

[151] Khodaei F Hosseini SM Omidi M Hosseini SF Rezaei M. Cytotoxicity of metformin against ht29 colon cancer cells contributes to mitochondrial sirt3 upregulation. J Biochem Mol Toxicol. (2021) 35:e22662. doi: 10.1002/jbt.22662, PMID: 33147367

[152] Potočnjak I Šimić L Vukelić I Batičić L Domitrović R. Oleanolic acid induces hct116 colon cancer cell death through the P38/foxo3a/sirt6 pathway. Chem Biol Interact. (2022) 363:110010. doi: 10.1016/j.cbi.2022.110010, PMID: 35690101

[153] Kang TS Yan YM Tian Y Zhang J Zhang M Shu Y. Yzl-51n functions as a selective inhibitor of sirt7 by nad(+) competition to impede DNA damage repair. iScience. (2024) 27:110014. doi: 10.1016/j.isci.2024.110014, PMID: 38947512 PMC11214487

